# Diffusion tensor imaging of white matter integrity and pain-related outcomes in chronic nonspecific neck pain

**DOI:** 10.1038/s41598-025-25303-x

**Published:** 2025-11-21

**Authors:** Rungtawan Chaikla, Kittichai Wantanajittikul, Marco Barbero, Deborah Falla, Munlika Sremakaew, Sureeporn Uthaikhup

**Affiliations:** 1 Department of Physical Therapy, Integrated Neuro-Musculoskeletal, Chronic Disease, and Aging Research Engagement Center (ICARE), Faculty of Associated Medical Sciences, Chiang Mai University, Chiang Mai, Thailand; 2 Department of Radiologic Technology, Faculty of Associated Medical Sciences, Chiang Mai University, Chiang Mai, Thailand; 3 Department of Business Economics, Health and Social Care, Rehabilitation Research Laboratory 2rLab, University of Applied Sciences and Arts of Southern Switzerland, Manno, Switzerland; 4 Centre of Precision Rehabilitation for Spinal Pain (CPR Spine), School of Sport, Exercise and Rehabilitation Sciences, College of Life and Environmental Sciences, University of Birmingham, Birmingham, UK

**Keywords:** Neck pain, White matter integrity, Fractional anisotropy, Mean diffusivity, Diffusion tensor imaging (DTI), Magnetic resonance imaging, Chronic pain

## Abstract

Chronic neck pain is associated with neuroplastic changes in brain structure, but its alterations in white matter integrity remain unclear. This study aimed to investigate white matter microstructural changes in patients with chronic nonspecific neck pain and their relationships with pain-related outcomes (i.e., pain duration, intensity, disability, extent and pressure pain thresholds (PPTs) over the neck). Using diffusion tensor imaging, fractional anisotropy (FA) and mean diffusivity (MD) were analyzed in 30 individuals with neck pain and 30 pain-free controls through whole-brain and region-of-interest (ROI) approaches. The results revealed that individuals with neck pain had lower FA and higher MD in several white matter tracts related to pain processing (e.g., corpus callosum, internal capsule, superior longitudinal fasciculus and superior corona radiata) compared to controls (adjusted-p values < 0.05). In specific ROIs, FA was negatively correlated with pain intensity, disability, extent and PPTs (*r*=-0.50 to -0.60, adjusted-p values < 0.05) while MD was positively correlated with pain duration, disability and extent (*r* = 0.54 to 0.59, adjusted-p values < 0.05). These results suggest widespread white matter alterations in people with chronic nonspecific neck pain, providing insights into altered central mechanisms that may contribute to pain persistence. However, the results should be interpreted with caution given the limitations of data acquisition.

## Introduction

Neck pain often manifests as persistent or recurrent symptoms^1^, yet its underlying mechanisms remain poorly understood. Central sensitization, maladaptive movement patterns and psychosocial aspects are thought to contribute to the persistence or recurrence of neck pain^2,3^. It has been suggested that chronic pain can induce structural changes in gray matter, particularly in areas involved in pain processing, such as the prefrontal cortex, anterior cingulate cortex, somatosensory cortex, insula and thalamus^4^. Additionally, pain may interfere with white matter connectivity, affecting the pathways responsible for transmitting pain signals^5,6^. These alterations provide valuable insight, suggesting that chronic pain is not merely a sensory experience but a complex, dynamic condition involving maladaptive neural plasticity and network dysfunction, which influence how pain is perceived, modulated and experienced over time^7^.

White matter consists of myelinated nerve fibers that connect neurons across brain regions, forming functional networks^8^. The white matter plays a crucial role in pain modulation, sensorimotor integration, cognitive processing and emotional regulation^9–11^. Alterations in white matter integrity, particularly in measures of fractional anisotropy (FA) and mean diffusivity (MD) metrics have been suggested to be associated with maladaptive neuroplasticity in individuals with chronic pain^12^. FA is an index of water diffusion directionality reflecting the structure of axonal cell membranes and myelin sheath tracts, while MD represents the overall magnitude of water molecule diffusion in all directions^13^. Lower FA and higher MD values may contribute to the persistence of pain by impairing neural mechanisms involved in pain modulation, motor control and emotional processing, thereby influencing pain perception and response^5,12,14^. Only one study has investigated alterations in white matter structures within regions of interest (ROIs), including the superior cerebellar peduncle, anterior and posterior corona radiata, internal capsule, cingulum (cingulate gyrus and hippocampus), fornix and stria terminalis, tapetum and splenium of the corpus callosum, in female patients with chronic neck pain^15^. The results showed that those with chronic whiplash-associated disorders exhibited decreased FA and increased MD in the left cingulum hippocampus and tapetum compared to both patients with chronic idiopathic neck pain and pain-free controls. There was no difference in white matter observed in patients with chronic idiopathic neck pain compared to controls. These findings may suggest that decreased FA and increased MD could be associated with the underlying cause of neck pain, whether of traumatic or non-traumatic origin. However, the study was limited by its use of a whole-brain analysis approach, which may have reduced its ability to detect more subtle or widespread white matter changes. In other chronic pain conditions, alterations in white matter integrity have been shown to correlate with pain outcomes such as pain duration, intensity and sensitivity^16–18^. The relationship between altered white matter integrity and pain characteristics in chronic neck pain remains unknown. Research on changes in white matter integrity in people with chronic neck pain is limited and requires further investigation. Understanding changes in white matter integrity would provide valuable insights into pain mechanisms and the potential for enhancing therapeutic strategies.

The aim of this study was to investigate FA and MD values using diffusion tensor imaging (DTI) technique, applying both whole-brain and ROIs analytic approaches, to examine brain areas involved in pain processing in individuals with chronic nonspecific neck pain compared to controls. Both whole-brain and ROI analyses are common approaches used in DTI studies to investigate brain microstructure. While whole-brain analysis provides a global perspective on white matter alterations, ROI analysis offers more targeted, region-specific insights^19^. The relationships between ROI-based FA and MD values and pain-related outcomes (i.e., pain duration, pain intensity, pain extent, neck disability and pain sensitivity over the cervical spine) were also explored. It was hypothesized that individuals with chronic nonspecific neck pain would exhibit lower FA and higher MD in widespread brain regions related to pain processing compared to pain-free controls. Altered FA and MD values within specific ROIs were also expected to correlate with pain-related outcomes.

## Materials and methods

### Study design

This cross-sectional study was conducted as part of a larger project, with participant recruitment occurring between January and November 2023 and took place at the research unit of the Departments of Physical Therapy and Radiologic Technology, Chiang Mai University. The study was approved by the Ethics Committee of the Faculty of Associated Medical Sciences, Chiang Mai University (No. AMSEC-63EX-101) and was conducted in accordance with the declaration of Helsinki. Written informed consent was obtained from all eligible participants prior to the commencement of the study. The study was reported according to the Strengthening the Reporting of Observational Studies in Epidemiology (STROBE) guidelines.

### Participants

Determining sample size for DTI studies is challenging due to variability in diffusion metrics, the complexity of image processing and the issue of multiple comparisons^20^. In this study, sample size was determined based on a systematic review investigating white matter integrity using DTI in chronic pain conditions^20^. Among 53 included studies comparing chronic pain patients with healthy controls, most had sample sizes of 11–30 participants per group and the median sample size across all studies was 23. Based on this range, a sample size of 30 participants per group was deemed a robust and suitable estimate for detecting meaningful between-group differences in DTI studies.

Thirty participants with chronic nonspecific neck pain (aged between 18 and 59) and 30 controls of similar age, gender and body mass index were recruited for the study. Participants were recruited through social media (e.g., Facebook and Instagram) and advertisements in the community, hospitals, physical therapy clinics and universities. Participants in the neck pain group were required to have chronic nonspecific neck pain (≥ 3 months) and an average neck pain intensity over the past week ≥ 35 mm on a 0–100 mm visual analogue scale (VAS)^21^. The control group was required to have no history of neck pain in the past year. Exclusion criteria for all participants were a history of head and neck injury or surgery, regular headaches (e.g., migraine and tension-type headaches), known or suspected vestibular pathology or dizziness caused by underlying pathology in the ear, brain and sensory nerve pathways, neurological or musculoskeletal conditions that could affect the outcomes (e.g., back pain, fibromyalgia and temporomandibular disorders), metabolic conditions, body mass index ≥ 25 kg/m^2^, psychiatric disorders (e.g., major depression and schizophrenia), contraindications to MRI (e.g., pregnancy/breastfeeding, claustrophobia and ferromagnetic implants). All inclusion and exclusion criteria were assessed using self-report questionnaires and interviews.

All potential participants in both groups were also screened for symptoms of anxiety and depression over the past week using the Hospital Anxiety and Depression Scale (HADS)^22^. Participants with a total score of less than 8 on either anxiety or depression subscale, indicating the absence of symptoms, were eligible for the study^22^.

### Questionnaires

#### Demographics

A general questionnaire was developed to collect participants’ demographic details and other relevant information.

#### Visual analogue scale (VAS)

A 0–100 mm VAS, ranging from 0 (no pain) to 100 mm (the worst pain imaginable) was used to measure neck pain intensity over the past week^23^. A higher score indicates greater pain intensity (no pain = 0–4, mild pain = 5–34 mm, moderate pain = 35–74 mm and severe pain = 75–100 mm)^21^. The VAS has been shown to have excellent reliability (Intraclass Correlation Coefficient, ICC = 0.97)^24^.

#### Neck disability index (NDI)

The NDI was used to assess disability in activities of daily living due to neck pain^25,26^. It consists of 10 items addressing pain intensity, personal care, lifting, reading, headaches, concentration, work, driving, sleeping and recreation. The NDI is scored on a scale of 0 to 50, where 0 indicates no disability and 50 represents complete disability (0–4 no disability, 5–14 mild disability, 15–24 moderate disability, 25–34 severe disability and ≥ 35 complete disability)^26^. The score can then be expressed as a percentage. The NDI has been shown to have good reliability (ICC = 0.85)^25^.

### Pain drawings – pain extent

A set of digitized pen-on-paper pain drawings were used to measure pain extent, according to previously validated procedures^27,28^. Participants were provided with a red marker and two coded male or female body charts (ventral and dorsal view) printed on A4 sheets. They were instructed to draw the extent and distribution of their pain symptoms over the last two weeks as accurately as possible, regardless of pain intensity and type. Participants were explicitly asked to avoid the use of circle outlines or cross-marks. Once pain drawings were completed, participants confirmed their satisfaction with the representation of their pain distribution.

For digitalization and analysis, a web service-based platform (Sketch Your Pain, https://syp.spslab.ch) was used to process the pain drawing datasets. Each pain drawing was scanned and uploaded as a Portable Document Format (PDF) file onto the platform, where an automated pain-spot recognition algorithm detected and analyzed the pain drawings^27^. Pain extent was calculated as the sum of pixels and expressed as a percentage of the total body chart area, with any shading outside the body chart borders excluded from the analysis.

The measurement of pain extent in individuals with neck pain has been shown to be reliable, with high test-retest reliability (ICC = 0.92, 95% CI: 0.87–0.98)^29^.

### Pressure pain thresholds (PPTs)

PPTs were measured using a digital pressure algometer (Somedic AB, Farsta, Sweden), as described previously^30^. PPTs were assessed bilaterally at the articular pillars of the C2-3 and C5-6 cervical segments, with pressure applied at a constant rate of 40 kPa/sec. Participants were instructed to press a button to release a switch as soon as the applied pressure was perceived as painful. Each site was measured three times and the mean values were used for analysis. The PPTs were performed by a physical therapist trained in administering PPT measurements over five 2-hour sessions. Intra-rater reliability was good to excellent (ICC = 0.88 at C2-3 and ICC = 0.92 at C5-6).

### White matter integrity

#### Diffusion tensor imaging acquisition

DTI data was conducted by an MR physicist using a Philips Ingenia 1.5 Tesla MR scanner (Philips Healthcare, Amsterdam, Netherlands) with a SENSE head coil. DTI images were acquired with a single-shot, spin-echo, echo-planar imaging (SE-EPI) with the following parameters: repetition time (TR) = 4065 ms, echo time (TE) = 86 ms, field of view (FOV) = 225 × 225 mm², voxel size = 2.5 × 2.5 × 2.5 mm³, flip angle = 90°, slice thickness = 2.5 mm and an acquisition time of 9.04 min. The DTI protocol included 32 diffusion-weighted images acquired along 32 non-collinear gradient directions (b = 1000 s/mm²), along with a single non-diffusion-weighted reference image (b0 = 0 s/mm²).

#### Diffusion tensor imaging analysis

DTI data was processed using the Functional MRI of the Brain (FMRIB)’s Diffusion Toolbox (FDT) within the Oxford Centre for Functional MRI of the Brain Software Library (FSL)^31^. The preprocessing pipeline included several steps including susceptibility-induced distortion correction (TOPUP), brain extraction (Brain Extraction Tool, BET), motion and eddy current correction (EDDY), diffusion tensor estimation (DTIFIT) and alignment of FA and MD maps using Tract-Based Spatial Statistics (TBSS)^32^. All acquired images were reviewed for potential artifacts, excessive motion and anatomical irregularities.

For TBSS analysis, FA maps were nonlinearly registered to a standardized 1 × 1 × 1 mm space (FMRIB58_FA) and then aligned to the MNI152 template using an affine transformation. To perform voxel-wise cross-subject statistical analyses, FA data were projected onto a mean FA skeleton representing the centers of each white matter tract in the brain. FA images from all participants were first aligned to a standard brain space using FMRIB’s Non-linear Imaging Registration Tool (FNIRT). A mean FA image was then generated and a projection technique was applied to create a mean FA skeleton, capturing the centers of major white matter tracts shared across participants. A threshold of 0.2 was used to define the skeleton, onto which each participant’s aligned FA data was projected. The skeletonized FA data underwent voxel-wise statistical analysis using FLS’s randomise tool, a nonparametric permutation-based inference method, with 5,000 permutations performed to assess group differences. Significant clusters were identified and corrected for multiple comparisons using Threshold-Free Cluster Enhancement (TFCE) approach. For visualization, significant regions were enhanced to improve the visibility of areas with low FA values in the skeleton. Additionally, thresholded TBSS results were thickened to local white matter tracts to facilitate a more precise anatomical interpretation of the findings. Beyond FA, TBSS was also applied to MD maps following the same analytical framework. To anatomically localize significant differences, the Johns Hopkins University (JHU) ICBM-DTI-81 white matter atlas was used to identify and label the affected white matter tracts.

For the ROIs analysis, mean FA and MD values were extracted from binary mask (JHU ICBM-DTI-81) of specific white matter tracts selected based on previous studies in chronic musculoskeletal pain conditions, specifically focusing on regions or tracts involved in transmitting information between brain areas related to pain perception, pain modulation, sensorimotor integration and emotional regulation^5,15,17,33^. These included superior longitudinal fasciculus, internal capsule (i.e., anterior and posterior limbs), external capsule, corona radiata (i.e., anterior, superior and posterior), cingulum (i.e., hippocampus and cingulate gyrus segments), corticospinal tract and thalamic radiation (i.e., anterior, superior and posterior).

### Statistical analysis

Participants’ demographics (i.e., gender, age, body mass index, hand dominance and HADS scores) and clinical characteristics (i.e., PPTs at C2-3 and C5-6) were compared between groups using an independent t-test for continuous variables and a Chi-square test for categorical variables.

TBSS was used to perform a whole-brain analysis of FA and MD values to compare differences between groups with TFCE approach applied to correct for multiple comparisons at a significant threshold of *p* < 0.05. The TFCE method enhances cluster-like structures in statistical images by simultaneously accounting for both signal intensity (height) and spatial extent (cluster size). It integrates cluster information across a range of thresholds, generating voxel-wise values that quantify the degree of cluster-like local support^34^.

A multivariate analysis of variance (MANOVA) was used to examine between-group differences in FA and MD values for the ROIs separately for each hemisphere. Multiple comparisons were corrected using the Benjamini-Hochberg (BH) procedure to control the false discovery rate (FDR) (adjusted p-value < 0.05)^35^. The BH procedure ranks all p-values from smallest to largest and determines a critical value for each based on its rank, the total number of tests and the predetermined FDR level^36^. Effect sizes were calculated using partial eta squared (η^2^_p_) to quantify the magnitude of between-group differences in FA and MD values, with values of 0.01, 0.06 and 0.14 interpreted as small, moderate and large effects, respectively^37^.

Pearson’s correlation coefficient was used to investigate the relationships between FA and MD values for each ROI and neck pain outcomes (i.e., pain duration, pain intensity, neck disability, pain extent and PPTs at C2-3 and C5-6). Multiple comparisons were also corrected for FDR using the Benjamini-Hochberg method (adjusted p-value < 0.05). Correlation coefficient values were interpreted as follows: 0.00–0.10 = negligible, 0.10–0.39 = mild, 0.40–0.69 = moderate, 0.70–0.89 = strong and 0.90–1.00 = very strong^38^. All statistical analyses were conducted using SPSS, version 22 (IBM Corporation, Armonk, NY).

## Results

### Demographics and clinical characteristics of participants

Table 1 presents the demographic and clinical characteristics of participants. One participant from the control group was excluded from the analysis due to a data acquisition error during DTI scanning. No differences in participants’ demographics were observed between the groups (*p* > 0.05). Both groups reported no symptoms of anxiety and depression. Participants with chronic neck pain experienced moderate neck pain intensity and mild neck disability and exhibited lower PPTs at C2-3 and C5-6 compared to controls (*p* < 0.001).

**Table 1:** Demographics and clinical characteristics of participants.

	Neck pain (*n* = 30)	Control (*n* = 29)
**Demographics**		
Gender (% female)	63.33	65.52
Age (years)	32.00 ± 9.56	31.96 ± 10.00
Body mass index (kg/m^2^)	20.83 ± 1.97	21.38 ± 2.00
Side of dominant hand (% right)	96.67	96.55
HADS - anxiety score (0–21)	4.33 ± 2.07	3.48 ± 2.03
HADS - depression score (0–21)	2.70 ± 2.57	2.27 ± 2.03
**Clinical characteristics**		
Side of neck pain		
Unilateral pain (n, left/right)	1/2	-
Bilateral pain (n, more painful side: left/right)	16/11	-
Pain duration (months)	30.50 ± 34.11	-
Pain intensity (VAS, 0–100 mm)	60.17 ± 12.53	-
Neck disability (NDI, 0–100)	22.00 ± 8.03	-
Pain extent (%)	3.48 ± 1.16	-
Pressure pain thresholds (kPa)		
C2-3	470.69 ± 153.15	664.76 ± 166.04*
C5-6	480.08 ± 129.87	676.37 ± 162.41*

Data are presented as mean ± standard deviation unless otherwise indicated. n = number of participants, HADS = Hospital Anxiety and Depression Scale, VAS = visual analog scale, NDI = Neck Disability Index, * = *p* < 0.001.

### Whole-Brain Tract-Based Spatial statistics (TBSS) analysis

**Fig. 1:**
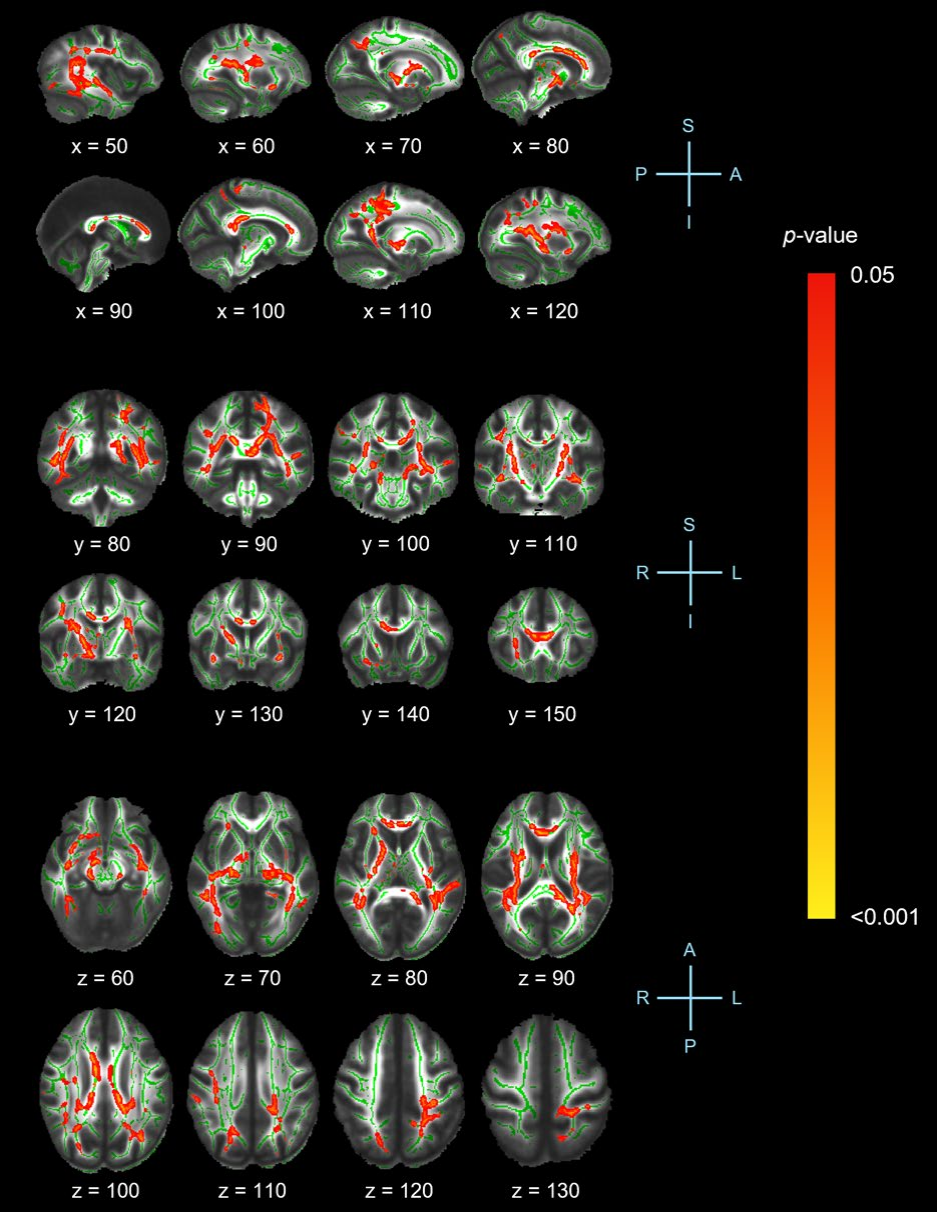
Tract-Based Spatial Statistics (TBSS) analysis showing significant differences in fractional anisotropy (FA) values between participants with neck pain and controls. White matter structures exhibit lower FA in the neck pain group compared to controls. Clusters are significant at *p* < 0.05, corrected for multiple comparisons using threshold-free cluster enhancement (TFCE). Statistical results are overlaid on the Montreal Neurological Institute (MNI152) template (grayscale) and the mean FA skeleton (green) for visualization. Red-yellow voxels indicate regions of significantly reduced FA. X, Y and Z-coordinates are displayed. A, anterior; P, posterior; L, left; R, right; S, superior; I, inferior.

Figure 1 displays the results of the whole-brain TBSS analysis of FA values between groups. Relative to controls, the neck pain group exhibited lower FA in the corpus callosum (body, genu and splenium), fornix, both hemispheres of the cerebral peduncle, corona radiata (superior and posterior), external capsule, uncinate fasciculus, inferior fronto-occipital fasciculus, superior longitudinal fasciculus, internal capsule (retrolenticular segment and posterior limb) and thalamic radiation (superior and posterior), left hemisphere of the cingulum adjacent to hippocampus and stria terminalis and right hemisphere of the anterior corona radiata, superior fronto-occipital fasciculus, anterior limb of the internal capsule and anterior thalamic radiation (TFCE-corrected *p* < 0.05).

**Fig. 2:**
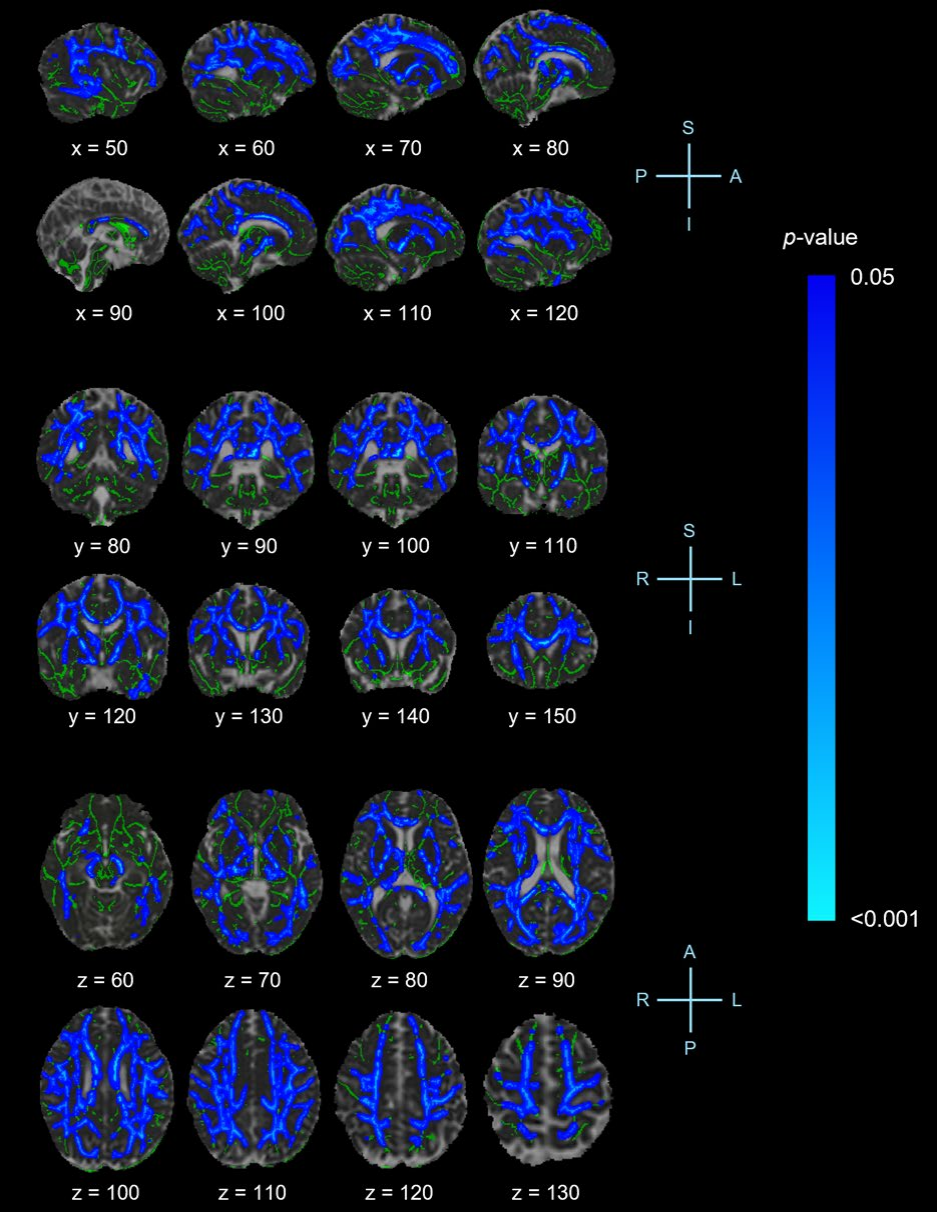
Tract-Based Spatial Statistics (TBSS) analysis showing significant differences in mean diffusivity (MD) values between participants with neck pain and controls. White matter structures exhibit higher MD in the neck pain group compared to controls. Clusters are significant at *p* < 0.05, corrected for multiple comparisons using threshold-free cluster enhancement (TFCE). Statistical results are overlaid on the Montreal Neurological Institute (MNI152) template (grayscale) and the mean MD skeleton (green) for visualization. Blue-light blue voxels indicate regions of significantly increased MD. X, Y and Z-coordinates are displayed. A, anterior; P, posterior; L, left; R, right; S, superior; I, inferior.

Figure 2 displays the results of the whole-brain TBSS analysis of MD values between groups. Relative to controls, those with chronic neck pain exhibited higher MD in the corpus callosum (body, genu and splenium), fornix, both hemispheres of the cerebral peduncle, corona radiata (anterior, superior and posterior), cingulum cingulate gyrus, external capsule, fronto-occipital fasciculus (inferior and superior), superior longitudinal fasciculus, internal capsule (retrolenticular segment, anterior and posterior limb) and thalamic radiation (anterior, superior and posterior) and right stria terminalis (TFCE-corrected *p* < 0.05).

There were no regions demonstrating higher FA or lower MD in those with neck pain compared to controls (TFCE-corrected *p* > 0.05).

### Regions of interest-based analysis

The MANOVA revealed significant differences between groups in FA values across the ROIs in the left hemisphere (*p* = 0.03, η_p_^2^ = 0.40). No differences between groups were observed in the right hemisphere (*p* = 0.84, η_p_^2^ = 0.15). After correction for multiple comparisons, those with chronic neck pain demonstrated significantly lower FA in the left hemisphere of the anterior limb of the internal capsule, posterior limb of the internal capsule, posterior corona radiata and superior corona radiata (adjusted p-values < 0.05) (Table 2).

**Table 2:** Fractional anisotropy (FA) for all regions of interest (ROIs) between participants with neck pain (*n* = 30) and controls (*n* = 29).

ROIs	Left hemisphere		Adjusted *p*-value	ES	Right hemisphere		Adjusted *p*-value	ES
	Control	Neck pain			Control	Neck pain		
SLF	0.42 ± 0.03	0.41 ± 0.03	0.46	0.03	0.44 ± 0.02	0.43 ± 0.03	0.57	0.05
ALIC	0.45 ± 0.04	0.42 ± 0.04	**0.04**	0.11	0.47 ± 0.03	0.46 ± 0.03	0.90	0.00
PLIC	0.58 ± 0.04	0.56 ± 0.03	**0.02**	0.14	0.57 ± 0.03	0.58 ± 0.02	0.87	0.00
EC	0.37 ± 0.03	0.36 ± 0.03	0.36	0.03	0.35 ± 0.03	0.34 ± 0.04	1.00	0.02
ACR	0.39 ± 0.03	0.38 ± 0.02	0.33	0.03	0.41 ± 0.02	0.40 ± 0.03	1.00	0.01
PCR	0.43 ± 0.02	0.41 ± 0.03	**0.04**	0.15	0.44 ± 0.02	0.42 ± 0.02	0.18	0.11
SCR	0.46 ± 0.02	0.44 ± 0.02	**0.02**	0.15	0.45 ± 0.02	0.45 ± 0.02	1.00	0.02
CgH	0.32 ± 0.06	0.33 ± 0.05	0.79	0.01	0.30 ± 0.06	0.29 ± 0.05	0.97	0.00
CgC	0.41 ± 0.06	0.42 ± 0.08	0.75	0.00	0.35 ± 0.04	0.34 ± 0.05	1.00	0.01
CST	0.42 ± 0.04	0.43 ± 0.10	0.97	0.00	0.42 ± 0.05	0.42 ± 0.10	0.87	0.00
ATR	0.38 ± 0.02	0.38 ± 0.02	0.69	0.01	0.37 ± 0.02	0.37 ± 0.02	0.92	0.00
PTR	0.51 ± 0.03	0.49 ± 0.03	0.41	0.03	0.51 ± 0.04	0.51 ± 0.03	1.00	0.00
STR	0.35 ± 0.02	0.35 ± 0.02	0.79	0.00	0.36 ± 0.02	0.36 ± 0.02	0.86	0.02

Data are presented as mean ± standard deviation. SLF = Superior longitudinal fasciculus, ALIC = Anterior limb of internal capsule, PLIC = Posterior limb of internal capsule, EC = External capsule, ACR = Anterior corona radiata, SCR = Superior corona radiata, PCR = Posterior corona radiata, CgH = Cingulum hippocampus, CgC = Cingulum cingulate gyrus, CST = Corticospinal tract, ATR = Anterior thalamic radiation, PTR = Posterior thalamic radiation, STR = Superior thalamic radiation, ES = effect size (partial eta squared, η^2^_p_).

The MANOVA revealed significant differences in MD values across the ROIs between groups on both the left (*p* = 0.002, η_p_^2^ = 0.50) and right (*p* = 0.04, η_p_^2^ = 0.39) hemispheres. After correction for multiple comparisons, those with chronic neck pain demonstrated significantly higher MD in both hemispheres of the superior longitudinal fasciculus, posterior limb of the internal capsule and superior corona radiata, the left hemisphere of the external capsule and the right hemisphere of the anterior limb of the internal capsule (adjusted p-values < 0.05) (Table 3).

**Table 3:** Mean diffusivity (MD) for all regions of interest (ROIs) between participants with neck pain (*n* = 30) and controls (*n* = 29).

ROIs	Left hemisphere		Adjusted *p*-value	ES	Right hemisphere		Adjusted *p*-value	ES
	Control	Neck pain			Control	Neck pain		
SLF	0.72 ± 0.02	0.73 ± 0.02	**0.01**	0.17	0.71 ± 0.02	0.72 ± 0.01	**< 0.001**	0.21
ALIC	0.70 ± 0.02	0.71 ± 0.01	0.33	0.04	0.71 ± 0.01	0.72 ± 0.01	**0.04**	0.11
PLIC	0.70 ± 0.02	0.71 ± 0.01	**0.01**	0.18	0.69 ± 0.01	0.70 ± 0.01	**0.02**	0.16
EC	0.75 ± 0.02	0.76 ± 0.02	**0.03**	0.13	0.72 ± 0.02	0.73 ± 0.02	0.12	0.07
ACR	0.74 ± 0.03	0.75 ± 0.02	0.65	0.01	0.75 ± 0.03	0.76 ± 0.02	0.52	0.02
PCR	0.76 ± 0.03	0.76 ± 0.03	0.52	0.02	0.79 ± 0.04	0.79 ± 0.03	0.54	0.01
SCR	0.70 ± 0.02	0.71 ± 0.02	**0.03**	0.12	0.70 ± 0.01	0.71 ± 0.01	**0.02**	0.15
CgH	0.86 ± 0.14	0.83 ± 0.04	0.46	0.03	0.90 ± 0.12	0.90 ± 0.10	0.97	0.00
CgC	0.76 ± 0.03	0.77 ± 0.04	0.45	0.02	0.76 ± 0.03	0.77 ± 0.03	0.29	0.04
CST	0.95 ± 0.16	1.00 ± 0.23	0.51	0.01	0.96 ± 0.16	1.01 ± 0.23	0.55	0.02
ATR	0.75 ± 0.02	0.75 ± 0.02	0.80	0.00	0.78 ± 0.04	0.78 ± 0.02	0.77	0.00
PTR	0.78 ± 0.04	0.79 ± 0.02	0.31	0.04	0.83 ± 0.07	0.82 ± 0.03	0.53	0.01
STR	0.90 ± 0.08	0.91 ± 0.08	0.51	0.01	0.90 ± 0.07	0.93 ± 0.07	0.18	0.06

Data are presented as mean ± standard deviation (x 10^−3^ mm^2^/s). SLF = Superior longitudinal fasciculus, ALIC = Anterior limb of internal capsule, PLIC = Posterior limb of internal capsule, EC = External capsule, ACR = Anterior corona radiata, SCR = Superior corona radiata, PCR = Posterior corona radiata, CgH = Cingulum hippocampus, CgC = Cingulum cingulate gyrus, CST = Corticospinal tract, ATR = Anterior thalamic radiation, PTR = Posterior thalamic radiation, STR = Superior thalamic radiation, ES = effect size (partial eta squared, η^2^_p_).

### Correlations between diffusion metrics (FA and MD) and pain outcome measures

**Fig. 3:**
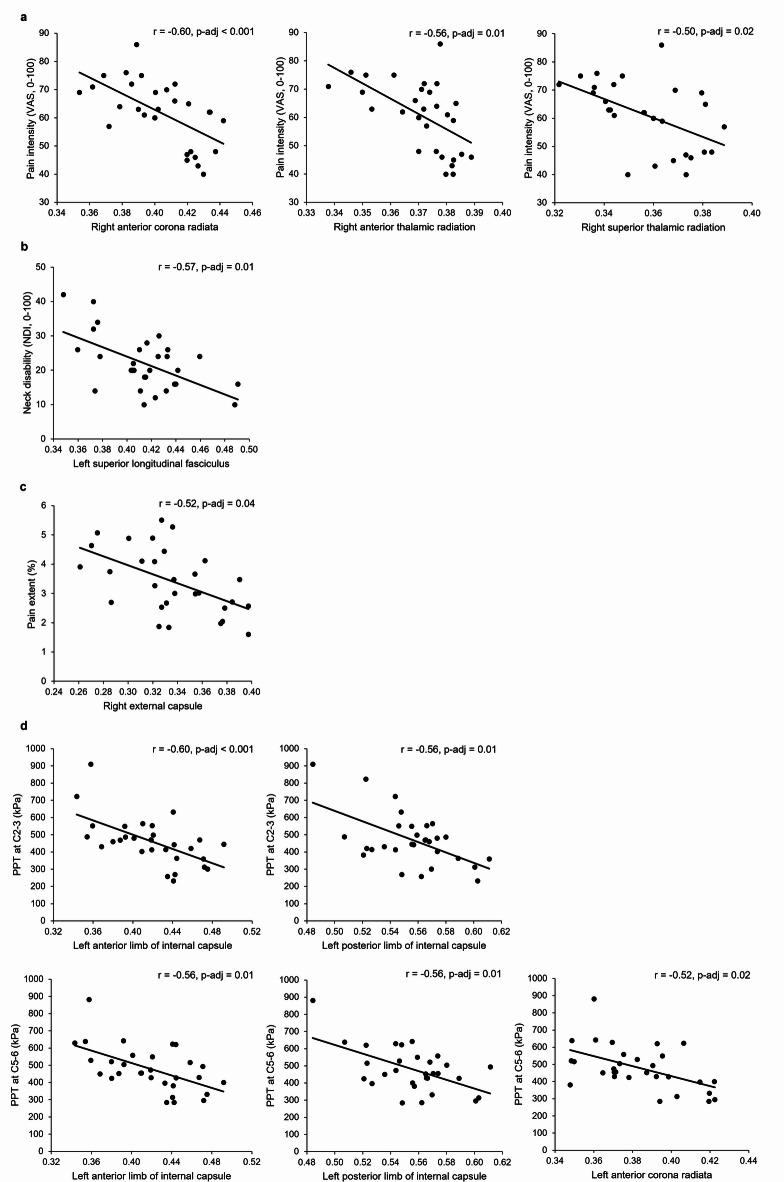
Significant correlations between fractional anisotropy (FA) values of regions of interest (ROIs) and pain-related outcomes in participants with neck pain (adjusted p values < 0.05): (**a**) pain intensity, (**b**) neck disability, (**c**) pain extent and (**d**) pressure pain threshold (PPT) at C2-3 and C5-6 levels.

There were negative correlations between neck pain intensity (VAS score) and FA in the right anterior corona radiata, anterior thalamic radiation and superior thalamic radiation (*r* = −0.50 to −0.60, adjusted p-values < 0.05) (Fig. 3a). Neck disability was negatively correlated with FA in the left superior longitudinal fasciculus (*r* = −0.57, adjusted p-value = 0.01) (Fig. 3b). Pain extent was negatively correlated with FA in the right external capsule (*r* = −0.52, adjusted p-value = 0.04) (Fig. 3c). PPTs at C2-3 and C5-6 levels were negatively correlated with FA in the left anterior limb of the internal capsule, posterior limb of the internal capsule and anterior corona radiata (*r* = −0.52 to −0.60, adjusted p-values < 0.05) (Fig. 3d). No correlation was observed between neck pain duration and FA values (adjusted p-values > 0.05).

**Fig. 4:**
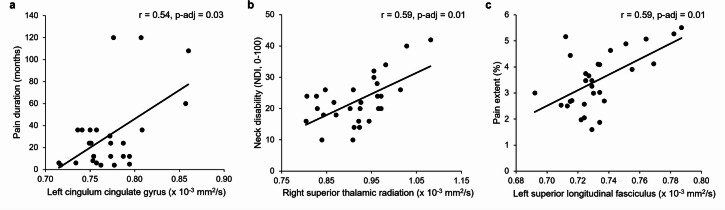
Significant correlations between mean diffusivity (MD) values of regions of interest (ROIs) and pain-related outcomes in participants with neck pain (adjusted p values < 0.05): (**a**) pain duration, (**b**) neck disability and (**c**) pain extent.

There was a positive correlation between duration of neck pain and MD in the left cingulum cingulate gyrus (*r* = 0.54, adjusted p-value = 0.03) (Fig. 4a). Neck disability was positively correlated with MD in the right superior thalamic radiation (*r* = 0.59, adjusted p-value = 0.01) (Fig. 4b). Pain extent was positively correlated with MD in the left superior longitudinal fasciculus (*r* = 0.59, adjusted p-value = 0.01) (Fig. 4c). MD values were not correlated with neck pain intensity and PPTs (adjusted p-values > 0.05).

## Discussion

This study demonstrates alterations in white matter integrity in people with chronic nonspecific neck pain compared to asymptomatic controls. Specifically, participants with chronic neck pain exhibited lower FA and higher MD values in various white matter tracts across multiple brain regions involved in pain processing, sensorimotor integration and cognitive and emotional function. Additionally, FA and MD values were related to pain-related outcome measures. These findings suggest widespread white matter microstructural abnormalities in people with chronic nonspecific neck pain, which may reflect disrupted communication between brain regions involved in pain perception and emotional responses, potentially contributing to the persistence of pain^39^.

White matter integrity is essential for optimal pain transmission and regulation^40^. In diffusion tensor imaging, FA and MD values are key metrics of the integrity of white matter in the brain. Generally, lower FA indicates reduced microstructural integrity of white matter, characterized by less directionally constrained water diffusion^13^. Conversely, higher MD suggests decreased tissue density or integrity, reflected by increased movement of water molecules within the tissue^13^. Together, lower FA and higher MD values reflect microstructural abnormalities in white matter, which may result from factors such as reduced axonal density, demyelination, disruptions in fiber organization and inflammation^13^. Such microstructural abnormalities in white matter have been observed in various conditions, including chronic musculoskeletal pain^12^. In this study, whole-brain analysis revealed that people with chronic neck pain exhibited lower FA and higher MD in several white matter tracts, including the corpus callosum, fornix, cerebral peduncle, corona radiata, external capsule, uncinate fasciculus, fronto-occipital fasciculus, superior longitudinal fasciculus, internal capsule, thalamic radiation, cingulum and stria terminalis. ROI-analysis further revealed that those with chronic nonspecific neck pain exhibited lower FA in the internal capsule, posterior corona radiata and superior corona radiata and higher MD in the superior longitudinal fasciculus, internal capsule, superior corona radiata and external capsule. The pathophysiological mechanisms responsible for microstructural alterations in white matter in people with chronic neck pain are not fully understood. However, such changes may be attributed to sustained neuronal and glial overactivation, leading to neuroinflammation, demyelination, reduced axonal density and disrupted fiber organization^41,42^. Persistent nociceptive input can also induce central sensitization, where hyperexcitable neurons undergo synaptic reorganization, amplifying pain signals and contributing to widespread abnormalities in brain connectivity^43^. Additionally, it was noted that all of the significant regions are integral to various functions, from motor control and sensory processing to emotion regulation, memory and cognition^5,44^. Specifically, the internal capsule modulates pain and transmits sensory signals from the thalamus to the postcentral gyrus through the corona radiata, crucial for sensorimotor communication and cortico-thalamic connections^45,46^, whereas the external capsule, connecting the limbic system with cortical regions, is involved in the emotional and cognitive aspects of pain regulation^47^. The superior longitudinal fasciculus, connecting the frontal lobe to the temporal and parietal lobes, integrates somatosensory and cognitive processes and is associated with chronic pain-related neuroplasticity (i.e., adaptations in brain networks contributing to persistence of pain)^48,49^. Thus, alterations in these pathways may indicate disruptions in neural mechanisms of pain processing, potentially influencing perception and modulation.

It has been suggested that altered white matter integrity may contribute to chronic musculoskeletal pain, acting as a cause, predisposing factor, consequence or compensatory adaptation^5^. Widespread white matter alterations have been demonstrated in patients with chronic musculoskeletal pain conditions^12^. In chronic neck pain, Coppieters et al.^15^ reported no changes in white matter integrity in patients with chronic idiopathic neck pain compared to controls. This finding is inconsistent with the results of the present study, which has demonstrated altered white matter integrity in patients with nonspecific neck pain. The discrepancy between our findings and those of Coppieters et al.^15^ may be attributed to differences in characteristics of neck pain (i.e., pain duration, intensity and disability) and data acquisition methodology (i.e., field strength and number of diffusion gradient directions). It has been suggested that higher field strength (i.e., 3.0 T) provides a higher signal-to-noise ratio and greater sensitivity, allowing more precise imaging of tissue diffusion properties^50^. However, given this discrepancy, the results of this study should be interpreted with caution.

There were significant correlations between FA and MD values in the selected ROIs and some pain-related outcomes. FA values in the anterior corona radiata, anterior thalamic radiation and superior thalamic radiation were negatively correlated with pain intensity whereas FA in the left superior longitudinal fasciculus correlated with neck pain disability and FA in the external capsule with pain extent. In other words, greater pain intensity, disability and pain extent were associated with lower FA in the specific regions. Besides, a higher MD in the specific regions (i.e., the cingulum cingulate gyrus, superior thalamic radiation and superior longitudinal fasciculus) was also associated with either longer pain, greater disability and a larger pain area. The regions significantly correlated with pain-related outcomes are primarily involved in cognitive processing, sensorimotor integration and pain modulation, all of which contribute to the perception and experience of pain^45,47,51^. Additionally, the result showed greater PPTs observed for those with chronic neck pain were correlated to lower FA values in the left internal capsule and anterior corona radiata. When essential pain-processing pathways are impaired (e.g., lower FA), the transmission or modulation of pain signals may become less effective^14^. Overall, the relationships may reflect abnormalities in sensory-discriminative processing and an altered affective response to pain. Our results are consistent with previous studies in patients with fibromyalgia^16,18^ and patients with chronic back pain^52^, which reported associations between altered white matter integrity (lower FA values) and longer pain duration, greater pain intensity and higher heat pain thresholds. However, the results are in contrast with a previous study in patients with idiopathic neck pain, demonstrating no relationship between regional white matter integrity and PPTs over a remote site (quadriceps muscle)^15^. A potential factor is likely to be variations in the methods and site used to assess pain sensitivity.

The overall study suggests alterations in white matter integrity in patients with chronic nonspecific neck pain compared to controls, which may provide some insight into the central mechanisms underlying the condition. This is especially noteworthy given that significant correlations were observed between changes in white matter integrity across various neural tracts and pain-related outcomes. These findings suggest that changes in certain neural tracts may play a crucial role in persistence of chronic nonspecific neck pain, potentially leading to more targeted and effective management strategies. However, several factors may contribute to alterations in white matter integrity, even though some, such as comorbid pain conditions, were controlled for in this study. Further research in this area is warranted.

The study’s limitations are acknowledged. Participants in this study were predominantly female. Medication and management for neck pain were not controlled, which may have influenced the study results. The use of a 1.5 T scanner may have limited detection of subtle white matter changes compared to higher-field strength scanners, such as 3.0 T^50^. Additionally, the cross-sectional study design of the study prevents the determination of causal relationships between white matter changes and chronic nonspecific neck pain. Future research using a longitudinal study design is needed to clarify the direction of these associations. Research using higher field strengths and more diffusion directions is also recommended to confirm these findings in people with chronic nonspecific neck pain. Studies with larger sample sizes should be conducted to further validate the current findings. Further, randomized controlled trials should investigate whether specific therapies, such as manual therapy and therapeutic exercise, can improve white matter integrity in individuals with chronic neck pain.

## Conclusion

People with chronic nonspecific neck pain had lower FA and higher MD in white matter tracts involved in pain processing, sensorimotor integration and cognitive and emotional functions compared to pain-free controls. Additionally, white matter integrity was found to be associated with neck pain characteristics and pressure pain sensitivity. These findings suggest widespread microstructural alterations in white matter pathways, which may contribute to the experience and persistence of pain.

## Data Availability

The data that support the findings of this study are available from the corresponding author upon reasonable request.
